# Giant parathyroid carcinoma: Diagnostic, difficulties and therapeutic strategies: A case report

**DOI:** 10.1016/j.amsu.2021.102919

**Published:** 2021-10-09

**Authors:** Laila Bouzayan, Mohamed Yassine Mabrouk, Hassane Ait Ali, Abdelali Guelil, Achraf Miry, Jabi Rachid, Amal Benani, Mohammed Bouziane

**Affiliations:** a Department of Visceral Surgery and Digestive Oncology A, Mohammed VI University Hospital, Oujda, Morocco; b Department of Anatomopathology, Mohammed VI University Hospital, Oujda, Morocco

**Keywords:** Parathyroid carcinoma, Hyperparathyroidism, Hypercalcemia, Parathyroidectomy, Case report

## Abstract

**Introduction:**

Parathyroid carcinoma is a very aggressive malignant tumor. It is mostly revealed by clinical primary hyperparathyroidism.

**Case presentation:**

We report a rare case of parathyroid carcinoma in a 61-year-old-male patient who presented with a painless right-sided cervical tumefaction of hard consistency associated with cervical lymphadenopathy. Cervical ultrasonography showed a right parathyroid mass with intimate contact with the homolateral thyroid lobe. A parathyroidectomy enlarged to the adjacent thyroid parenchyma with a selective neck dissection level VI was performed.

**Clinical discussion:**

The clinical presentation is most often manifested with clinical features of primary hyperparathyroidism associating bone disorders. Surgery remains the treatment of choice. The benefit of adjuvant treatments is controversial and remains to be evaluated.

**Conclusion:**

Parathyroid carcinoma is a rare tumor. This rare entity is often presented with clinicobiological features of severe primary hyperparathyroidism.

## Highlights

• Parathyroid carcinoma is a very aggressive malignant tumor.
• The final diagnosis is made upon pathology study.
• The surgical resection must be “en bloc” and complete R0 including the tumor and ipsilateral thyroid lobe with selective cervical dissection level VI.
• The prognosis depends on the risk of local or distant recurrences and the control of hypercalcemia.

## 1 Introduction

Parathyroid carcinoma is a very aggressive and rare malignant tumor of the parathyroid gland, with an estimated prevalence of 0.005% of all cancers [1]. It is responsible for 0.4–5.2% of the causes of hyperparathyroidism [2]. The clinical and biological findings are not specific unlike the parathyroid adenoma making the diagnosis difficult. The treatment is based on en-bloc complete surgical resection [3]. The prognosis depends on the presence or not of local or distant recurrences and also the management of hypercalcemia.Through this case report, we highlight the main clinical, histological, and therapeutic characteristics of this rare clinical entity. This case has been reported following the SCARE criteria [4].

## 2 Case presentation

A 61 –year- old male patient with a medical history of chronic kidney failure under no specific treatment was admitted to the emergency department for the management of diffuse bone pains and altered generation health status.

Physical examination revealed a stupurous, irritable and pale patient with a Glasgow score of 13 15.

Cervical examination revealed a painless right-sided cervical tumefaction of hard consistency associated with cervical lymphadenopathy.

Laboratory analysis showed increased serum concentrations of parathyroid hormone (PTH; 1507pg/mL [normal range, 15–65 pg/mL]) and calcium 185mg/L [normal range, 86.00–100.00 mg/L]), albumin 33mg/l (normal range, 35–50 mg/l) with a renal failure, creatinine 17.30(normal range,6–12 mg/l), a moderate inflammatory syndrome (white blood cells at 16,850 and C reactive protein at 54) and normochromic normocytic anemia (hemoglobin 7.8 (normal range, 13–18 g/dl), mean corpuscular volume 78.90fl (normal range 80–98 fl), mean corpuscular hemoglobin concentration 28(normal range 32–36%).

After stabilization of the patient in the intensive care unit, cervical ultrasonography showed a right parathyroid mass with intimate contact with the homolateral thyroid lobe suggestive of suspicious lymphadenopathy.

CT-fused MIBI scintigraphy revealed a right ovoid tissular retro thyroid mass, suggesting a parathyroid lesion.

**Fig. 1:**
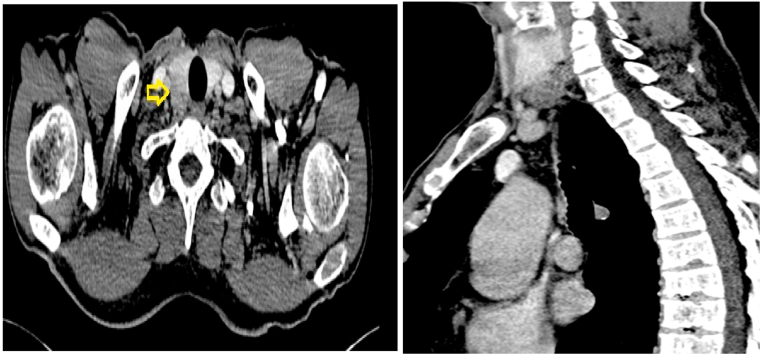
(A and B) Cervical computed tomography scan showing a hypodense retro-thyroid mass (arrow). A: axial section. B: sagittal section.

The cervical, thoracic, and abdominopelvic scan a parathyroid mass located in the lower right parathyroid gland, associated with multiple intraparenchymal renal and non-obstructive bilateral calcic stones, multiple osteolytic areas of the peripheral and axial skeleton (Fig. 1A and B).

**Fig. 2:**
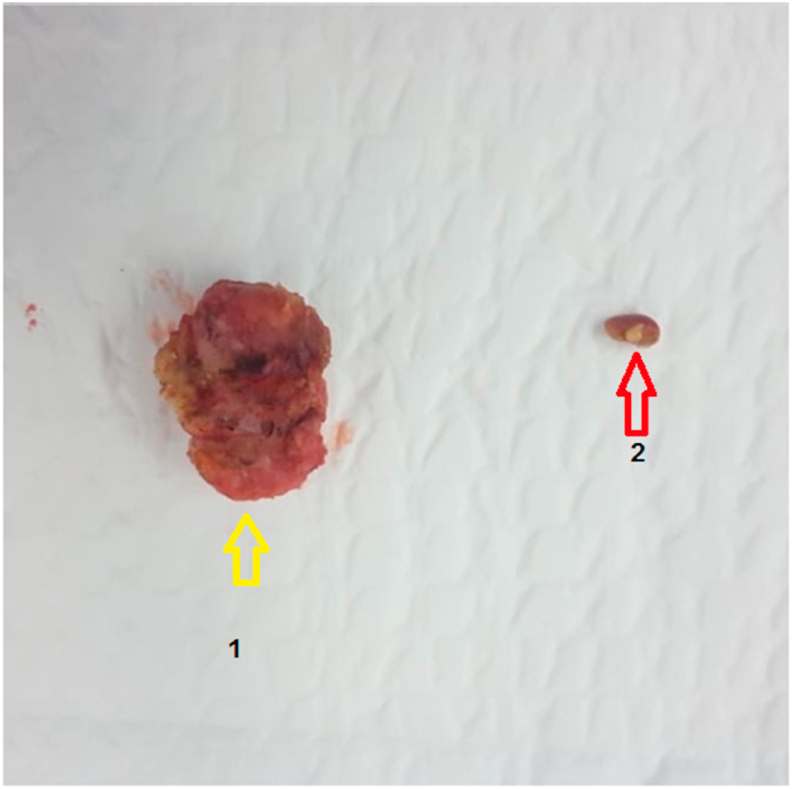
Image showing 1: Right inferior parathyroid (yellow arrow) 2: enlarged nodule (red arrow) at thyroidal parenchyma. (For interpretation of the references to color in this figure legend, the reader is referred to the Web version of this article.)

The patient underwent a parathyroidectomy enlarged to the adjacent thyroid parenchyma with a selective neck dissection level VI (Fig. 2) which was performed by our experienced surgical professor at the university teaching hospital.

The pathological study revealed a parathyroid neoplasm of probable malignancy suggesting a parathyroid carcinoma.

**Fig. 3:**
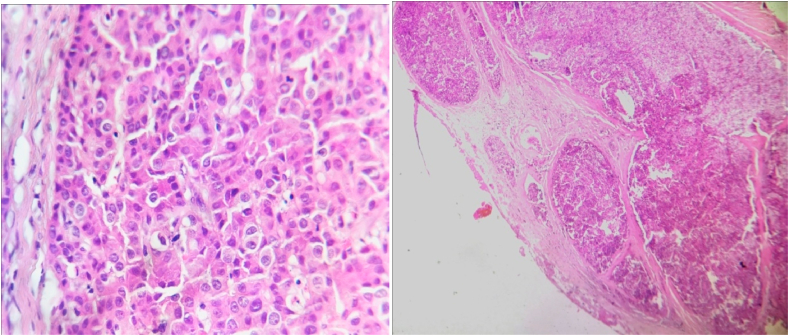
A: Microphotography showing moderate atypia in tumor cells, with many observed mitoses (HE; 400X). B: Microphotography showing vascular invasion (HE, 100X). HE: hematoxylin and eosin.

The tumor proliferation has intimate contact with the thyroid parenchyma without clear invasion (Fig. 3A) with vascular emboli and perineural invasion (Fig. 3B).

The postoperative outcomes were uneventful without any complications and the patient was discharged from the hospital on postoperative day 3.

At a 6-month follow-up, the patient is asymptomatic with normal serum calcium.

## 3 Discussion

Parathyroid adenocarcinomas are rare tumors accounting for 0.4–5.2% of the causes of primary hyperparathyroidism [2]. The diagnostic is often difficult and requires the confrontation of the clinical, biological, the macroscopic aspect during resection and the pathology confirmation.

These tumors affect both men and women, with a median age of 50 years.

The clinical presentation is most often manifested with clinical features of primary hyperparathyroidism associating bone disorders: osteoporosis and fibrocystic osteitis, renal: urolithiasis and renal failure, digestive system: pain, nausea, acute pancreatitis, cardiac disorders: arterial hypertension and arrhythmias [5]. Malignant parathyroid disease is suggested by the presence of a palpable cervical mass - which contrasts with benign parathyroid tumors - as well as the presence of cervical lymphadenopathy or laryngeal paralysis.

The laboratory test is characterized by the presence of severe hypercalcemia with values greater than 3.5 mMol/L, hypophosphatemia, a high level of alkaline phosphatase, and hyperchloremic metabolic acidosis. On the other hand, a serum level below 4 times the normal is not very suggestive of the diagnosis. Faced with a clinical-biological picture suggestive of primary hyperparathyroidism, radiological exploration should systematically include cervical ultrasonography which will objectify a hypoechoic retro thyroid paratracheal cervical mass, with irregular contours and signs of invasion of adjacent structures [6].

The ultrasonography must be completed by Tc99m-Sestamibi scintigraphy - sensitive to 91% - which highlights the presence of a parathyroid tumor (hyper fixation) and localizes it [6]. These two Standard investigations confirm the parathyroid nature of the cervical mass and look for the presence of any cervical lymphadenopathy.

If there is a strong suspicion of malignancy, thoracic abdominal-pelvic CT scans for distant metastases should be performed [7].

Surgery remains the treatment of choice. The surgical procedure must obey the oncological resection principles and must be “en bloc” resection of the tumor gland and ipsilateral thyroid lobe (parathyroidectomy + ipsilateral loboisthmectomy), respecting the recurrent nerve if it is not invaded, as well as ipsilateral mediastinal-recurrent lymph node dissection of principle (group VI) [8]. If metastatic lymphadenopathy is present, a functional ipsilateral cervical lymph node dissection will be performed. In the event of incomplete resection, the risk of recurrence is high, and an enlarged tumor resection is the best option to hope for a cure [9,10].Given the high risk of recurrence in the event of incomplete resection, enlarged tumor excision remains the best option for optimizing the prognosis. [11,12]

Macroscopically parathyroid carcinoma affects only one gland and appears as a firm mass of grayish color that measures on average 3 cm in diameter and weight between 2 and 10 g [13,14]. In general, this mass is poorly limited, adhering to adjacent tissues. In addition, the tumor can sometimes be encapsulated which makes it indistinguishable from an adenoma. (13.)

Microscopically, the architecture is typically trabecular, but we can observe aspects in rosettes and diffuse layers. The main aspects suggesting a carcinoma are the presence of fibrous bands, high mitotic activity, invasion of the capsule and/or soft tissues as well as a vascular and peri-nervous invasion [15].

Recently, immunohistochemical techniques have revealed a significantly higher Ki-67 labeling in carcinomas compared to adenomas and hyperplasias with a threshold value varying from 40 to 80 labeled cells per 1000. It should be noted that the study of P53 and Bcl2 proteins are of no interest for differential diagnosis [16,17].

Postoperative monitoring includes research, in the post-intervention recovery room and the hospital room, for dysphonia, cervical hematoma, or a hemorrhage.

Biological monitoring is adapted on a case-by-case; it includes at least a phosphocalcic balance, magnesemia, an ionogram, and an evaluation of renal function. The frequency of carrying out biological examinations is adapted to each patient, knowing that the balance sheet normalizes in most cases from the third day.

Parathyroid carcinoma is radio-resistant, external radiotherapy directed to the neck after surgery is indicated in the case of R1 surgery to destroy the remaining tumor cells and thus reduce the risk of recurrence [18].

Chemotherapy is usually ineffective and its main goal is to achieve remission, although this is often short-lived. There are no standard cytotoxics for treating parathyroid cancer. Some agents used, alone or in combination, include Dacarbazine (DTIC), 5-fluorouracil (5-FU, Adrucil), Cyclophosphamide (Procytox), methotrexate, doxorubicin (Adriamycin), and lomustine (CeeNU, CCNU) [18]. Recurrences occur in 45% of cases. Metastases are observed in around 30% of cases [19], they are mainly lymph nodes, then pulmonary, hepatic, and bone. The 5-year and 10-year overall survival rates are 85% and 49%, respectively [18].

Mortality is most often secondary to a complication of hypercalcemia. Overall survival is not influenced by tumor size or the presence of metastases. Finally, our patient was satisfied with our medical and surgical management procedure.

## 4 Conclusion

Parathyroid carcinoma is a rare tumor. This rare entity is often presented with clinicobiological features of severe primary hyperparathyroidism with an initial calcium level greater than 3.0 mmol/l and a parathyroid lesion greater than 3 cm. The diagnosis of parathyroid carcinoma is made usually upon pathological study; the presence of tumor emboli, invasion of adjacent tissues, or metastases suggests malignancy. The surgical treatment with en-bloc R0 resection associated with selective neck dissection is the gold standard. The benefit of adjuvant treatments is controversial and remains to be evaluated.

## Ethics approval

No ethical approval was necessary.

## Sources of funding

The author(s) received no financial support for the research, authorship and/or publication of this article.

Dr Bouzayan Laila: Have written the article, have consulted the patient, and participated in the surgery.

Dr Mabrouk Mohamed Yassine: have helped writing the article, data collection.

Dr Ait Ali Hassane, Dr Guellil Abdelali: supervised the writing of the manuscript.

Dr Merry Achraf: Interpretation of histological data.

Pr BenaniAmal (anatomopathology professor): confirm the histological diagnosis.

Pr Jabi Rachid: supervised the writing of manuscript.

Pr Bouziane Mohammed (oncology surgery professor): have supervised the writing of the paper, and has been the leader surgeon of the case.

## Research registration

Not available.

## Guarantor

Dr Bouzayan Laila, Pr BOUZIANE Mohammed.

## Consent of the patient

Written informed consent was obtained from the patient for publication of this case report and accompanying images. A copy of the written consent is available for review by the Editor-in-Chief of this journal on request.

## Provenance and peer review

Not commissioned, externally peer-reviewed.

## Declaration of competing interest

The authors declared no potential conflicts of interest concerning research, authorship, and/or publication of the article.
